# Floating photovoltaics performance simulation approach

**DOI:** 10.1016/j.heliyon.2022.e11896

**Published:** 2022-11-25

**Authors:** Sofiane Kichou, Nikolaos Skandalos, Petr Wolf

**Affiliations:** Czech Technical University in Prague, University Centre for Energy Efficient Buildings, 1024 Třinecká St. 27343 Buštěhrad, Czech Republic

**Keywords:** Floating photovoltaics (FPV), Solar tracking, FPV design, Energy yield, Techno-economic analysis

## Abstract

Floating photovoltaics (FPVs) provide various benefits especially where land is scarce (e.g., reducing land occupancy, water evaporation and environment control…), or when they are combined with hydropower plants (enhanced capacity factor and green energy generation). Software such as PV∗SOL, SAM and PVSyst® are commonly used for the design and simulation of land-based photovoltaic (PV) systems. However, when it comes to the simulation of photovoltaics installed on water surface, such software does not offer the option to directly simulate FPV systems.

In this work, a new approach combining MATLAB and Rhino/Grasshopper environments is proposed for the assessment of FPV systems performance. The approach is divided into various steps considering major influencing parameters such as temperature, irradiance, albedo, PV modelling, panel rows spacing, tilt angle, as well as the benefits of including a tracking mechanism. The proposed approach was validated against PV∗SOL simulations for land-based PV systems with a small deviation of less than 2.4%. FPVs simulations considering climatic conditions of Štěchovice, Czechia, showed an increase of the performance in the range of 3% compared to terrestrial PVs. This result is in accordance with some published studies based on real FPVs installations. Finally, the developed approach was applied in the simulations of two large-scale FPV systems with different designs (fixed and with a tracking mechanism) including economical aspects.

## 1 Introduction

Floating photovoltaic systems (FPVs) gained popularity in 2007 since the first commercialized power plant installed by SPG Solar in Napa Valley reservoir, California, USA. The FPV industry has showed a rapid grow during last few years with a total capacity exceeding 3 GW in 2020 [1]. The interest on FPVs is still persistent worldwide as, in the year 2020, approximately 2.6 GW of the total FPVs capacity projects were either under construction or fully functional [2]. The big share of FPV system installations are in Asian countries, however, the share of FPVs for the rest of the world is also increasing [2]. This FPVs share growth can be attributed to the various benefits they can offer e.g., resolve land-use conflicts, reduce water evaporation, enhance energy production, as well as, the ability to mitigate the greenhouse gas emissions and climate change concerns [3].

Large reserves and basins of water can be found all over the world, as well as they can accumulate in depleted mining areas. Therefore, installing PV systems on water surfaces can help save land and resolve the land-use conflicts between the solar PV sector and other fields – such as agriculture, buildings and industry [4]. Water surfaces can provide better microclimatic conditions for PV panels due to lower temperatures and higher windspeeds above their surfaces compared to land. Several research studies have demonstrated that the energy yield of FPVs is superior to that of ground-mounted and rooftop PV installations [5, 6, 7, 8], and this is mainly attributed to cooling effects of water bodies. Detailed thermal analysis presented in [9] demonstrated lower PV module temperatures of 5 °C depending on the chosen boundary conditions [10]. Therefore, lower operating temperatures make FPVs competitive in the southernmost regions, where the irradiance and the ambient temperature values are the highest.

Coupling FPVs with other Renewable Energy Systems (RESs) such as hydroelectric power plants (HPPs) can be advantageous and increases the capacity factor of the whole hybrid system. It is reported in [1] that covering 3–4% of the water area of some large HPP with FPV could double the projected solar installation capacity. Furthermore, the combination of FPVs and HPPs dispatch could mitigate the volatility of the PV production while making better use of existing transmission assets – a gain that could be particularly valuable in countries/regions with poor grids [1]. The partial coverage of water surfaces (basins) with FPVs has additional benefits such as reducing the rate of water evaporation which is crucial in locations where water is scarce [11, 12, 13]. The study published in [14], showed that the FPV system can reduce the evaporation from a wastewater pond in Iran by up to 70%.

The profitability of FPVs compared to ground PVs was analysed in [15]. Results indicated that FPVs can significantly support the renewable shift of the Spanish electricity market. By covering 1% of the reservoir surface, FPVs could generate the equivalent of 1.7% of the current national electricity demand. However, the author concluded that FPVs should reduce their Capital Expenditure (CAPEX) by up to 10% to be competitive with the ground PV plants [15]. In addition, the combination of offshore wind parks with FPV systems increases the cable usage (cable pooling), and can be beneficial in terms of technical and economic aspects [16].

Modelling and simulation are fundamental to understand the overall feasibility of FPVs in terms of techno-economics and environmental impacts. To the best of our knowledge, there is a lack of simulation tools which are dedicated to FPV modelling, performance assessment and analysis. So far in literature, FPVs performance assessments were based on conventional ground PV modelling and simulation tools/software, where several modifications are required. For instance, the System Advisory Model (SAM) was used by McKuin et al [17] to understand the techno-economic analysis of water-based PV systems in California to power pumping systems. Song and Choi [18] analysed the potential of FPV systems on a mine pit lake in Korea using SAM to conduct energy simulations based on local weather data and the system design. Nevertheless, it was found that the software underestimated the electrical output of the FPV system compared to the measured data of a real installation [19]. As an alternative, PVSyst was used to estimate the energy yield of FPVs composed of conventional [20] or bifacial [21] modules, as well as when combined with hydroelectric plants [22]. Combination of software was another option applied by the authors in [23] for the design of FPV installation in Livorno, Italy. PVSyst® was used for the analysis of losses due to shadings, and the Photovoltaic Geographical Information System (PVGIS) was used for the comparison and inclusion of tracking systems. Finally, Manoj Kumar et al. compared simulation results from three software (PVSyst®, SAM and Helioscope) with real measured data of a 2 MW FPV plant and reported the electricity generation deviation ranging from 18.4 to 38.6% [24]. Overall, the available PV simulation tools are mainly conceived for conventional land-PV installation.

Therefore, the aim of this work is to help in developing an alternative approach for the simulation and assessment of floating PV systems performance. The approach combines two different software; MATLAB is used for the modelling of PV outputs and data analysis, while Rhino/Grasshopper environment is used for the 3D modelling of the FPVs geometry and detailed solar radiation and shading analysis. The major influencing parameters for the simulation of the FPV performance – such as module temperature, albedo effect, panels spacing and module inclination – are described and studied. In addition, the effect of using a vertical-axis tracking mechanism is evaluated in terms of electricity generation and economical savings.

The paper is organized as follows. Section 2 presents the approach and relevant simulation models used for the calculation of the power output of a floating photovoltaic system, including solar irradiance and PV module temperature. Section 3 presents the effect of each design parameter (tilt angle, module spacing, etc.) and the potential energy gains. Finally, section 4 presents the implementation of the approach and techno-economic analysis of the FPV systems in continental climate conditions of Czechia.

## 2 Materials and methods

PV∗SOL, SAM and PVSyst® are few of the existing software widely used for the design and simulation of photovoltaic (PV) systems. However, none of them offers the option (without key assumptions to be made) to directly simulate and assess the performance of floating photovoltaic systems. In this work, an approach for the simulation and evaluation of FPVs is developed using MATLAB and Rhino/Grasshopper environments. The approach takes into consideration the major influencing parameters such as temperature, solar radiation, and the PV system configuration.

### 2.1 Albedo effect/irradiance

Albedo is a measure of a surface's potential to reflect the solar radiation. A default value of 0.2 is commonly used in PV yield simulation software as a good approximation for ground reflectivity. However, albedo varies throughout the day and seasons, and such assumption may lead to overestimations of irradiance and PV module's temperature. A model for estimating albedo of a water surface as a function of the sun position is given in Eq. (1) [22]:

(1) $$\rho =c^{r\;\sin\;\beta +1}$$

Where, c is the colour coefficient, r is the roughness coefficient, and β is the solar height. In this study, coefficients for lakes with clear water ripples were adopted from [25]. The sun angles of the selected location were obtained using the sun-path component [26] and the local climate file in Grasshopper for the calculation of the hourly albedo. Afterwards, results were passed to the irradiance model (using Daysim/Radiance simulation engine [27, 28]) for the calculation of incident solar radiation in each time step. FPV geometry with relevant optical surface properties and radiance parameters were also feeding the irradiance model.

**Figure 1:**
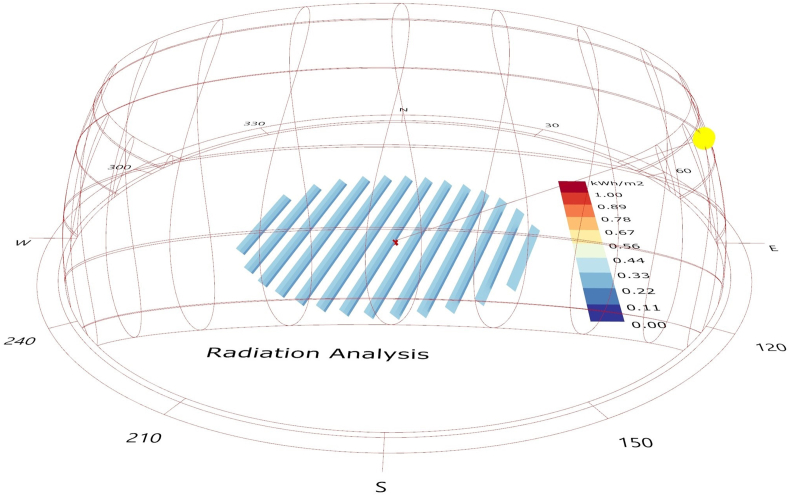
An example of the radiation analysis for the tracking system and PV modules position for 23 August at 8am.

Subsequently, simulations were performed considering different tilt angles to assess their effect on the PV generation for both fixed and tracking PV system. For the case of the one-axis tracking system, a separate model was prepared for the adjustment of the PV modules according to the sun position (azimuth variation) and calculation of the solar radiation (and associated shading) on the plane-of-array every time step (Figure 1).

### 2.2 Temperature effects

Another parameter with a significant effect on the FPV system output is the module temperature. An increase of the module temperature results in lower module efficiency, and thus the PV electricity generation. The linear relation between the electrical efficiency (*ƞ*) and the module temperature is represented by Eq. (2) [29]:

(2) $$\eta =\eta_{STC}\left[1-\gamma \left(T_{m}-T_{STC}\right)\right]$$

where ƞ_*STC*_ and *γ* are the electrical efficiency and temperature coefficient of the selected PV technology, respectively. *T*_*m*_ is the PV module operating temperature. *T*_*STC*_ is the PV module operating temperature at standard test conditions.

Considering the importance of the device temperature on the PV efficiency, a model that correlates the temperature of a PV module to the ambient conditions (ambient temperature (*T*_*a*_), solar radiation (*G*) and wind speed (*WS*) was used according to Eq. (3) (Table 1).

**Table 1:** Empirical models for the calculation of the PV operating temperature.

Reference	PV Type	Temperature model
[30]	Land	(3) $$T_{PV}=3.81+0.0282G+1.31T_{a}-1.65WS$$
[31, 32]	Floating	(4) $T_{FPV}=0.943T_{a}+0.0195G-1.528WS+0.3529$
[33]	Floating	(5) $T_{FPV}=2.0458+0.9458T_{a}+0.0215G-1.2376WS_{w}$
[33]	Floating	(6) $T_{FPV}=1.8081+0.9282T_{a}+0.215G-1.221WS_{w}+0.0246T_{w}$

FPV installations are often attributed to lower operating temperatures leading to higher efficiencies compared to land-based PV installations. However, cooling effect depends on the design configuration and several other parameters as they are stressed out in a recent literature review [15]. In a study by Hammoumi et al [34] FPV lower temperature was justified due to the lower water temperature compared to air. In another study [35], this temperature drop was attributed to the natural air convection occurring under the FPV module and to the presence of cooler ambient temperature above water. Generally, in an air-cooled FPV system, lower air temperature and higher wind speed are both influential parameters. In this regard, correlations between ambient and water temperatures [36] as well as the wind velocity on water with respect to the one on land [37] have been proposed in literature (Eqs. (7) and (8)) as follows:

(7) $$T_{w}=5+0.75\;T_{a}$$

(8) $$WS_{w}=1.62+1.17\;WS$$

where *T*_*w*_ is the water temperature °C, *T*_*a*_ is the ambient air temperature °C, *WS*_*w*_ is the wind speed on water and *WS* the wind speed on land (m/s). Then, three additional models (Eqs. (4), (5), and (6)) validated with measured data from real FPV systems were used for comparison and further analysis.

### 2.3 PV panel spacing

**Figure 2:**
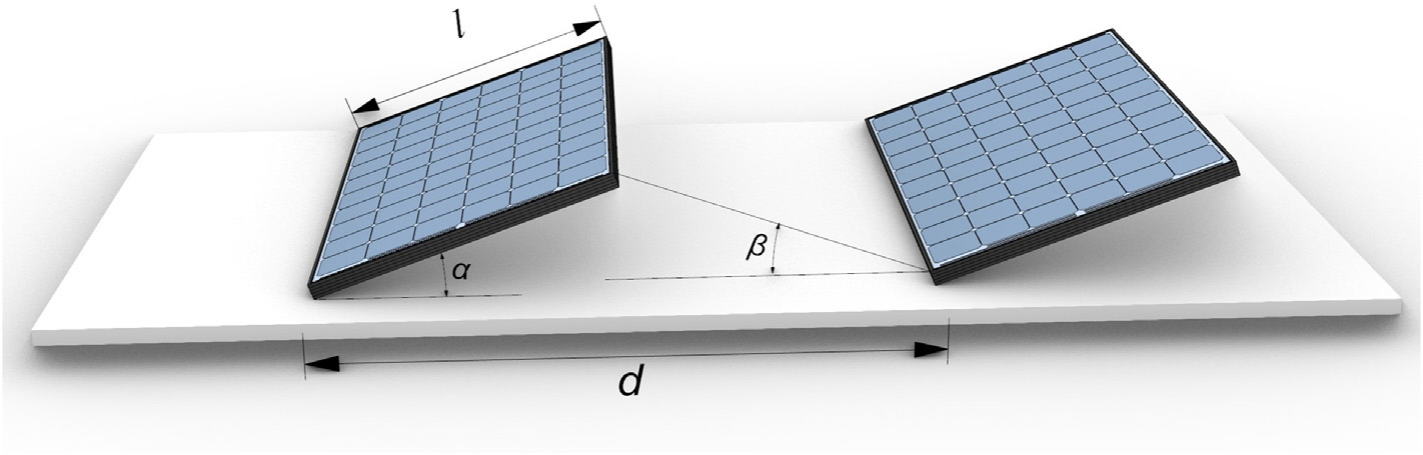
Schematic for the PV module spacing calculation.

Mutual shading of PV panels can have a significant effect on the performance of FPVs plants. Therefore, PV rows spacing is particularly important to avoid self-shading and maximize performance. A proper spacing allows for the maximization of the installed capacity and for the efficient use of the available area. The adequate space between the PV rows can be calculated based on the parameters given in the schematic shown in Figure 2. The width of the PV panel is represented by the variable *l*. The variable *α* represents the tilt-angle of the panel, and the variable β represents the sun angle at which no shading is casted, while *d* represents the distance between the rows’ edges. The simplest mathematical approach to determine the distance between the rows when they are facing south is based on the following equation (Eq. 9):

(9) $$d=\left(\frac{\sin\;\alpha }{\tan\;\beta }+\cos\;\alpha \right)l$$

### 2.4 PV modelling

The PV modelling was based on the one-diode five-parameters solar cell model. It includes a parallel combination of a photogenerated controlled current source *I*_*ph*_, a diode, described by the well-known single-exponential Shockley equation, a shunt resistance *R*_*sh*_ and a series resistance *R*_*s*_ modelling the power losses. The I–V characteristic of a solar cell is defined by the implicit and nonlinear expression given in Eq. (10).

(10) $$I=I_{ph}-I_{o}\left(e^{\left(\frac{V+R_{s}I}{nV_{t}}\right)}-1\right)-\left(\frac{V+R_{s}I}{R_{sh}}\right)$$

where *I*_*o*_ and *n* are the reverse saturation current and the diode ideality factor respectively. *V*_*t*_ represents the thermal voltage.

The selected solar cell model is widely used in the modelling and simulation of PV modules of different technologies (both crystalline and thin-film), and it provides a good compromise between complexity and accuracy. A detailed description of the model with the necessary equations to be used for the scalability of the model from solar cell to PV array can be found in [38].

**Figure 3:**
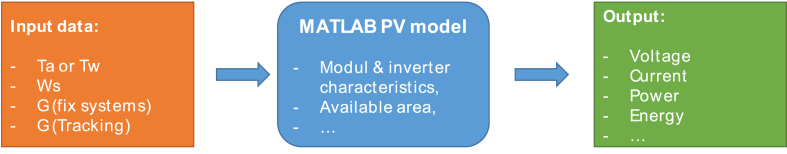
FPV model structure prepared in MATLAB environment.

The FPV model prepared in MATLAB environment has the general structure given in Figure 3. The electrical outputs necessary for the assessment of the FPV systems performance are determined based on location weather data and system characteristics. The FPV module temperature is calculated based on the models described in Table 1, while the plan of array solar irradiance (*G*)— corresponding to fixed FPV or FPV with a tracking system — is directly taken from simulations carried out in Rhino/Grasshopper environment (described in section 2.1). The input parameters related to PV module characteristics, inverter efficiency and the available water area are introduced to the model for the final design and sizing of the FPV system. Angular, spectral and temperature losses were considered in the model, while an appropriate performance ratio (PR = 0.87) was used to characterize the losses from DC to AC conversion (including mismatch, ohmic, inverter and wiring losses) [39, 40]. Finally, the outputs of the model can be hourly profiles of current and voltage as well as other metrics related to energy generation and yields of the PV system.

## 3 Results and discussions

The developed approach was tested for the location and semi-continental climatic conditions of Štěchovice, Czechia, the characteristics of which are given in Table 2. In addition, the results from most influencing parameters on the performance of the FPVs are presented and discussed in this section.

**Table 2:** Climate characteristics of the selected location.

Koppen climate zone	Temperate, No Dry Season, Warm Summer (Cfb)
Average annual temperature	9 °C
Annual total solar radiation	1073 kW h/m^2^
Average annual wind speed	3 m/s

### 3.1 Solar irradiance & tilt angle effect

The albedo values were calculated according to relative sun heights obtained from the sun-path diagram of the selected location. As expected, albedo is higher for low sun angles (morning/afternoon time) and lower for high sun angles (noon). Water surface reflectivity during daytime peak radiation ranges between 0.05 – 0.11 in summer and winter time, respectively. On average, it was found to be 0.098 which is below the default value (for the ground) of *ρ* = 0.2 used in commercial PV software. This albedo difference decreases solar radiation on plane of array and thus the power generation gains, while its effect depends on the selected PV tilt angle. The decrease of solar irradiance was found to be less than 7%, which is in agreement with other studies [3]. Detailed results for various tilt angles are given in Table 3.

**Table 3:** Albedo effects on solar irradiance for different tilt-angles.

Tilt angle	Reduction of solar irradiance on water compared to land
15°	-6.17%
25°	-3.25%
35°	-1.02%

**Figure 4:**
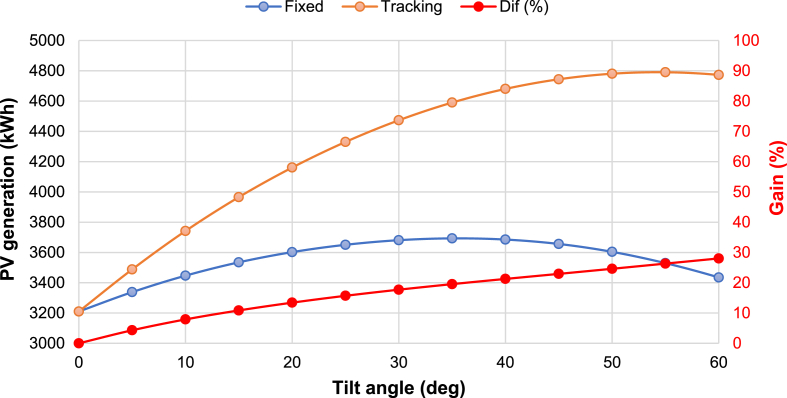
Tilt angle effects on selected FPV systems and potential gain when tracking system is used.

Results from the performance analysis of both a) fixed and b) tracking system are presented in Figure 4. For fixed systems, the optimal tilt angle in the studied location was found to be equal to 35° (15% higher potential compared to horizontal). In the case of PV systems with one-axis tracking system, the effect of the tilt angle is much more significant. For the optimal tilt angle of 55°, PV generation increases by 49% compared to horizontal. In addition, a comparison between fixed and tracking system is presented in Figure 4 (red line). An increase in the PV tilt angle results in an increase in the solar gains when tracking system is used. Considering the same PV system, this gain could be up to 30%. However, before final decisions are made, results should be considered together with the optimal spacing of the modules, since higher tilt angle leads to higher mutual shading of the arrays.

### 3.2 Temperature effect

**Figure 5:**
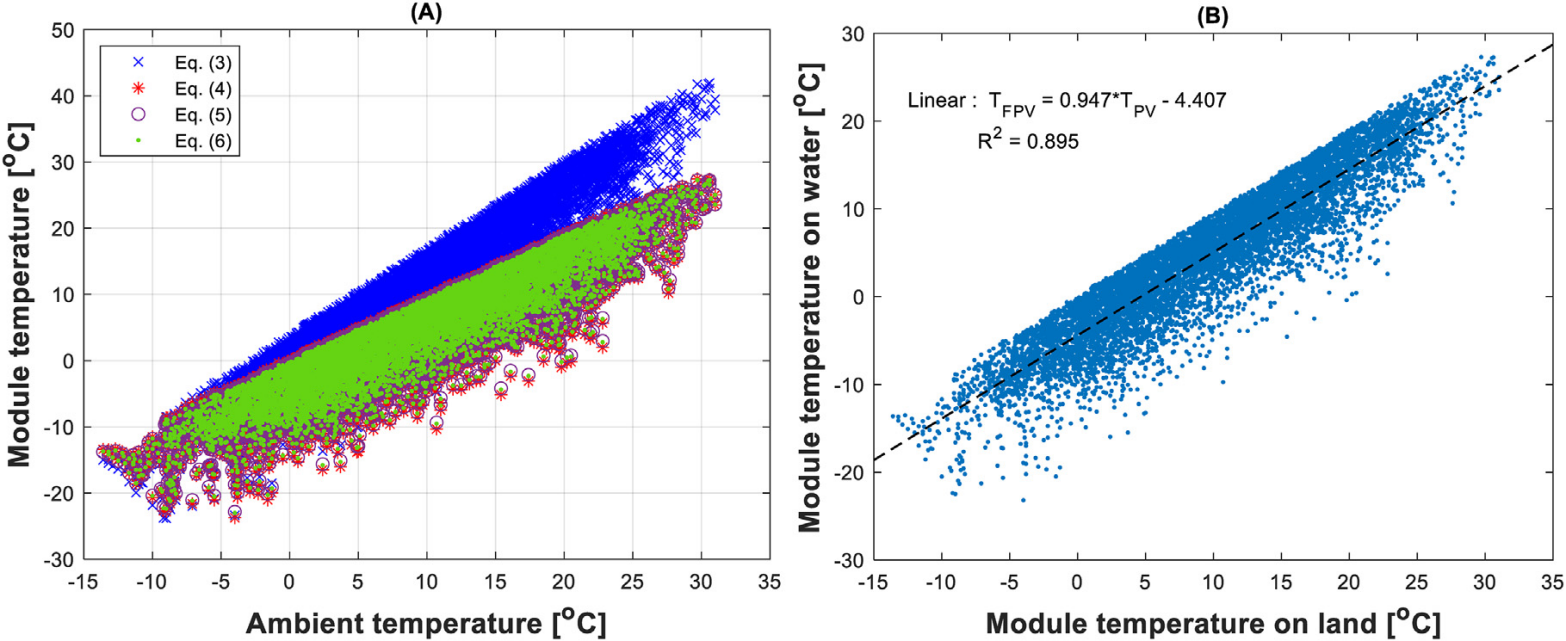
(a) comparison between module temperature installed on land and module temperature installed on water. (b) Extracted equation from module temperature on water based on Eqs. (4), (5), and (6).

The FPV module temperature was calculated based on the weather data of the selected location using models described previously (Table 1). Figure 5a shows the variation of the PV module temperature with respect to the ambient air temperature. It can be observed that the model used for the calculation of the PV module temperate (Eq. 3) leads to higher and different results compared to the rest of the models (Eqs. (4), (5), and (6)) dedicated to FPVs. The empirical equations (Eqs. (4), (5), and (6)) taken from previous research work provide similar trends even though they were obtained for FPVs installed in different locations. Figure 5b shows the linear correlation equation extracted from the combination of the three FPV model equations (Eqs. (4), (5), and (6)). From the obtained equation (Eq. 11) it may be concluded that the average difference between land PV module temperature (*T*_*PV*_) and FPV module temperature (*T*_*FPV*_) is approximately 5 °C. This result is in accordance with other research work present in the literature [8, 41].

(11) $$T_{FPV}=0.947\;T_{PV}-4.407$$

However, FPVs of different configuration and floating structure technology can experience significantly different thermal behaviour. The proposed approach can be easily adapted for the calculation of FPV temperature based on other models and experimentally validated U-values as reported in recent publication [42].

### 3.3 PV panel spacing

**Figure 6:**
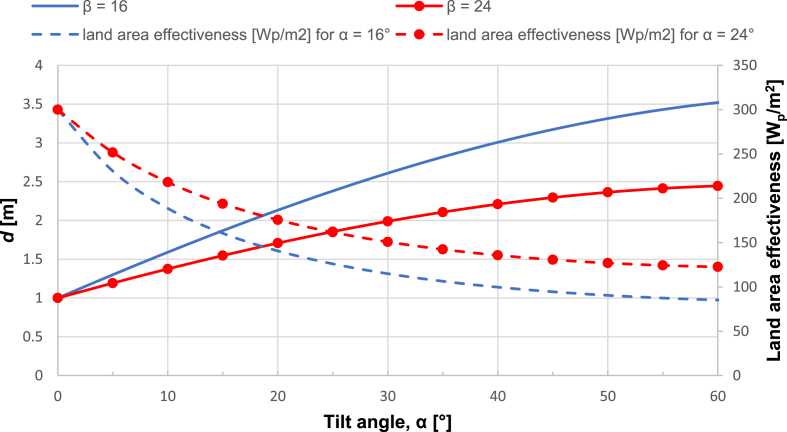
Sun-angle (β) effects on the spacing distance (*d*) and land area effectiveness.

The land area effectiveness represented in installed capacity by square meter (W/m^2^) can be increased by considering a slightly bigger sun-angle values. Parametric analysis carried out in Rhino/Grasshopper indicated that the sun-angle of 24° provides a good compromise between self-shading and performance of the PV systems. The obtained results showing the sun-angle effects on the spacing of the PV panels with standard dimensions (*l* = 1m) using Eq. (9) are given in Figure 6. By selecting a 30° inclination and sun-angle β = 16° (sun angle at which no shading is casted), the obtained land area effectiveness equals to 115 W/m^2^, however, for a sun-angle of β = 24° and same inclination, the land area effectiveness increases up to 150 W/m^2^. Such selection leads to different shading patterns within the day, the effect of which was determined by the solar irradiance model and considered in the detailed PV modelling.

### 3.4 FPV modelling and simulation

The developed PV model implemented in MATLAB software was firstly validated by comparing the simulation outputs with PV∗SOL software based on three ground-mounted PV systems (with and without a tracking mechanism). The considered PV systems have different tilt angles (15°, 25° and 35°) and they are composed of mono-crystalline PV modules the characteristics of which are listed in Table 4. The PV modules are installed on a defined land area of 10 m long by 10 m width, and the PV rows are spaced accordingly, based on the selected tilt angles. Table 5 summarizes the configuration of the three different PV systems.

**Table 4:** Selected PV module.

Module technology	Mono-crystalline
Max. power (W)	425
MPP Voltage (V)	42.5
MPP Current (A)	10.01
Open Circuit Voltage (V)	49.8
Short Circuit Current (A)	10.67
Module Efficiency (%)	20.5
Tem. Coeff. Pmax	-0.36 %/°C
Tem. Coeff. Voc	-0.26 %/°C
Tem. Coeff. Isc	0.02 %/°C

**Table 5:** Simulated PV systems.

	PV system 1	PV system 2	PV system 3
Tilt angle (°)	15	25	35
Land surface (m^2^)	100	100	100
Size (kW)	8.5	6.8	6.8
PV surface (m^2^)	41.5	33.2	33.2
Configuration (N_p_xN_s_)	5 × 4	4 × 4	4 × 4

**Figure 7:**
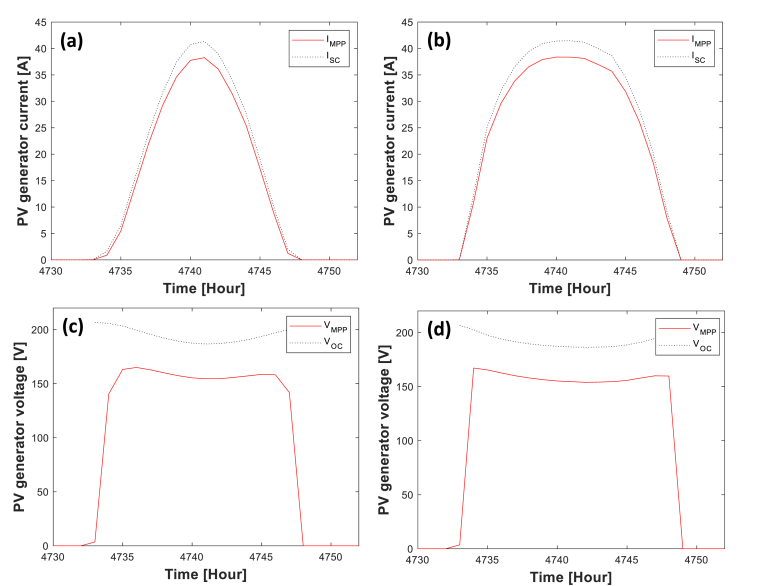
One day simulated current and voltage profiles related to PV system 2. Figure parts (a) and (c) correspond to fix FPV installation, while (b) and (d) refer to FPV with one-axis tracking mechanism.

Figure 7 shows the electrical outputs of *PV system 2* obtained from the developed MATLAB/Grasshopper model, while the detailed results from the comparison with PV∗SOL software are given in Table 6.

**Table 6:** Comparison between the developed PV model simulation results and PV∗SOL results.

	PV system 1		PV system 2		PV system 3	
Installation type	Fix	Tracking	Fix	Tracking	Fix	Tracking
PV size (kW)	8.5		6.8		6.8	

**Simulation results from PV∗SOL software**						
Annual Yield (kWh/kW)	1021.25	1084.45	1045.34	1178.8	1054.29	1247.44
Annual AC energy (kWh/year)	8681	9218	7108	8016	7169	8483
Tracking gain (%)	6.19		12.77		18.32	

**Simulation results from MATLAB model**						
Annual Yield (kWh/kW)	996.5	1061.4	1033.5	1167.9	1046.7	1245.7
Annual AC energy (kWh/year)	8470.1	9021.58	7027.5	7941.9	7117.6	8470.7
Tracking gain (%)	6.51		13.0195		19.01	

**Calculated deviation between the two software**						
AMAE (%)	2.43	2.13	1.13	0.92	0.72	0.15

There is a good match between the PV performance obtained from PV∗SOL and the developed model. The calculated absolute mean average error (AMAE) value for the annual AC generated electricity is less than 2.5%. The benefits from adding a tracking mechanism to the PV system are almost identical for both software. The PV gains due to tracking mechanism increase with the increase of the tilt-angle, and it is in accordance with results presented in section 3.1. For instance, for the *PV system 1* with 15° tilt-angle, the annual yield is around 1000 h, and this value can be increased by 6.51% if a tracking system is added. However, for the other systems it can be doubled (case of *PV system 2*) or tripled (case of *PV system 3*).

After the validation process, the FPV model - including all influencing parameters discussed above - was used for the simulation and the assessment of the three PV systems (described in Table 5) when installed on water. Additionally, the three PV systems were simulated considering two types of installation: fixed and with a vertical axis tracking mechanism. The obtained results are summarized in Table 7.

**Table 7:** FPV simulation results using our developed model.

	PV system 1		PV system 2		PV system 3	
Installation type	Fix	Tracking	Fix	Tracking	Fix	Tracking
PV capacity (kW)	8.5		6.8		6.8	
Annual Yield (kWh/kW)	1032,05	1100.56	1067.4	1209.28	1075.94	1285.98
Annual AC energy (kWh/year)	8772.4	9354.8	7258.1	8223.1	7316.4	8744.6
Gain from land to water (%)	3.57	3.69	3.28	3.54	2.8	3.28
Tracking gain on water (%)	6.64		13.3		19.52	

The comparison between floating and land PV installations is represented by the values “gain form land to water” given in Table 7. Representative gain range between 2.8% - 3.69% for the climatic conditions of the selected location, depending on the selected tilt angle and tracking system. The obtained gain is lower compared to similar studies dealing with the assessment of real FPV installations published in the literature. Gain values around 10% were reported in [43, 44], as well as a gain value greater than 10% was found in [45]. However, the authors of another case study from the Netherlands stated a similar gain of 3% [46].

Comparing the climatic condition, it is reasonable to expect that the benefits of installing PVs on water is higher (8–12%) in warm climates, as reported in [23]. However, other parameters related to system design (floating structure, selected tilt angles, water bodies, etc.) are equally important and could also affect the performance of the FPV systems.

The interest in tracking-type floating PVs has raised in the last years. It was shown that tracking systems can generate around 20% more energy compared to fixed floating PV [47]. The obtained results given in Table 7 are in the same range of the conclusions reported in literature. The greatest gain is obtained for FPV systems with the highest tilt-angle (35°) considered in the simulations [3].

## 4 Large-scale FPV system design and assessment

Results on the optimal design parameters were used for feasibility assessment of a large-scale FPV plant installation in Homole, Štěchovice under semi-continental climatic conditions of Czechia. Designs including a) fixed FPV and b) tracking FPV systems are presented and compared considering both energy and economical aspects.

### 4.1 Floating structure analysis and design

**Figure 8:**
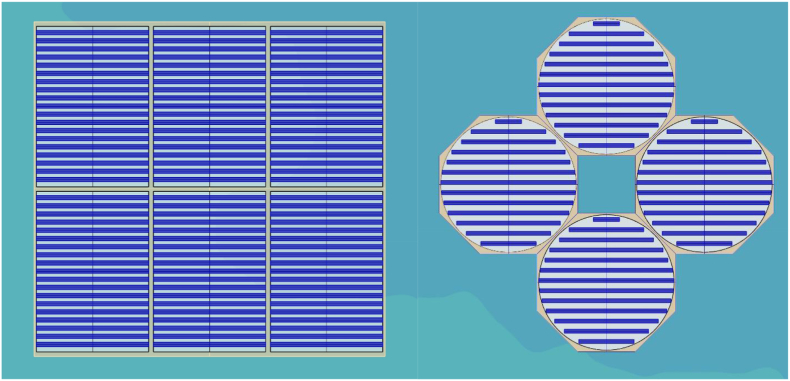
FPV configuration considering; fixed system with rectangular shape and 20° PV tilt angle and tracking FPV system with hexagon shape and 35° tilt angle.

The floating structure should firmly support the photovoltaic modules and provide sufficient resistance to external forces such as wind loads and waves. Moreover, it should secure long-term durability against corrosion, fatigue, etc. The total area considered for the design and installation of the FPV system on the lake corresponds to a rectangle of 60 m × 52 m. Two types of FPV systems design were evaluated as presented in Figure 8. The first design is related to a fixed FPV system, and the second one corresponds to an FPV system with a tracking mechanism.

The geometry of the tracking FPV system is based on the creation of fixed anchored polygons which at the same time form a service platform (Figure 8). Circles with a diameter of 24 m are inscribed in the polygons and are formed by a guide tube profile with a diameter of 100mm. The guide tube profile is connected to the polygon platform by a lattice structure. The platform is supported on the water surface by means of cylindrical plastic floats. PV modules are anchored in plastic floats and the circular geometry allowing the panels to rotate behind the sun is secured by reinforcing ropes, which are anchored to the polygon via a guide-tube profile. The anchor point is equipped with a moving mechanism that ensures the rotation of modules. With a design wind speed of 25 m/s the inclination of the modules was assumed to be in the range of 20–35° and the power requirement of the rotating mechanism is min 3 kW.

The fixed system assumes the same construction of plastic boxes carrying the photovoltaic panels. The geometry of the fixed system is secured by reinforcing ropes, which are provided with an anchoring system at their end. In both variants, the anchoring is provided by steel chains and concrete blocks.

Design configuration has an impact on the effective PV area as shown in Table 8. Selection of FPV system with tracking mechanism reduces the available area on water by 21%, mainly due to the circular geometry which facilitates the rotation of the floating platform. On the other hand, the surface losses for the fixed FPV system are reduced and counted to be approximately 8%. In this case, surface losses are due to free spaces in the edges and corridors for operation and maintenance (O&M) activities.

**Table 8:** Considered surface area for the installation of FPVs.

	FPV (Tracking)	FPV (Fix)
Available water surface area (m^2^)	3118	
Floating platform area (m^2^)	2858	3118
Effective area for PV installation (m^2^)	2461.7	2880.6
Surface losses due to the design (%)	21	7.6

The tilt-angle of the FPV system has direct effects on the FPV size and electricity generation. To evaluate these effects, the two considered FPV designs (fix and one-axis tracking mechanism) were simulated based on different tilt-angles. The tilt angles (10° and 20°) were selected for the fix FPV system based on some already available commercial floating structures [16]. In the case of FPV systems with a tracking mechanism, three tilt-angles (15°, 25° and 35°) were assumed for the evaluation of the different FPV scenarios.

Table 9 summarizes the simulation results obtained for the different FPV system configurations based on the selected tilt-angles. It is clearly shown that for all FPV scenarios, the higher the tilt-angle is, the better the yields are, represented by kWh/kW ratio. For the FPV system with tracking mechanism, the increase of the tilt-angle from 15° to 35° decreases the PV capacity (196 P V modules less) and enhances the system yield (kWh/kW) by 15.8%. This is in line with the values given in Table 7, where the comparison between the *PV system 3* and *PV system 1* annual yields was found to be 16.8%. The small deviation between the two values calculated from Tables 8 and 7 is mainly related to the system size and design. It can also be concluded, from the results given in Tables 6 and 7, that the annual electricity generation from FPVs with tracking system and 35° tilt-angle is 22.8% higher compared to fixed-land PV systems with the same characteristics (size and tilt-angle). On the other hand, FPV systems with high tilt-angles require a solid and stable mounting structure, and this will be reflected on the cost of the installation.

**Table 9:** Simulation of FPVs based on the selected mono-crystalline PV panel.

	FPV (tracking)			FPV (fix)	
Tilt angle (°)	15	25	35	10	20
Number of panels	792	676	596	912	760
FPV capacity (kW)	336.6	287.3	253.3	387.6	323.1
FPV energy (MWh/y)	359.2	335.1	313.2	379.2	333
Yield (kWh/kW)	1067.2	1166.6	1236.4	978.4	1030.6

The fixed FPV system with 20° tilt-angle has an enhanced yield (+5%), however, the annual energy generation is lowered by 12.2% compared to FPV system with 10° tilt-angle. This is mainly due to the reduced PV capacity (152 modules less). The choice of the tilt-angle for the fixed FPV system is mainly dependent of the economic analysis (section 4.2).

**Figure 9:**
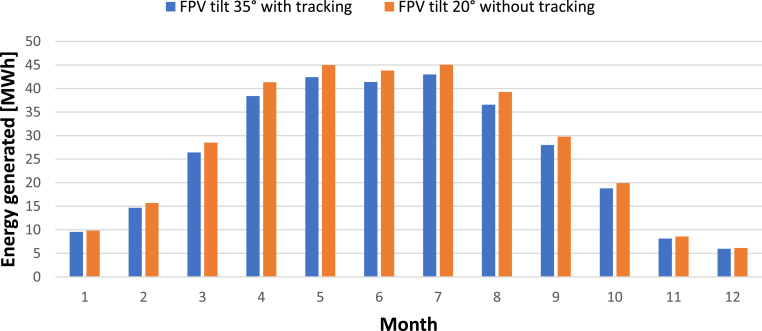
Monthly FPV energy generation related to the best scenarios of FPV systems with and without tracking mechanism.

Figure 9 compares the two best theoretical scenarios according to Table 9. The first scenario corresponds to the FPV with tracking and panels tilted 35°, and the second scenario represents the fixed FPV system with panels tilted 20°. It can be observed that both systems have similar monthly energy generation. Peak generation occurs during summer months, and it is found to be around 45 MW h, while it drops significantly during winter period, with a minimum generation of 5.9 MW h in December. Reduced capacity of tracking by 69.7 kW compared to fix FPV system, confirms the potential benefits of such systems when installed with higher tilt-angle.

### 4.2 Economic assessment

The FPV seems to be the right solution for a quick expansion of the PV sector. The cost of FPV is near to that of land-based solutions and the availability of large water surfaces is very considerable. A financial analysis was undertaken to compare the cost-effectiveness of the proposed design and the cases of a) fixed and b) tracking PV systems, respectively. The selected indicators were the Net Present Value (NPV) and the Discounted Payback Period (DPP). The former – defined as the sum of present incoming (benefits) and outgoing cash flows over the lifetime of the project – was calculated according to Eq. (12).

(12) $$NPV=-C+\sum_{t=1}^{N}\frac{F\left(t\right)}{{\left(1+i\right)}^{t}}$$

where *C* is the initial investment costs (€), *F(t)* is the annual income calculated as the difference between incoming and outcoming cashflows generated by FPVs (€/year), *N* is the lifetime of the investment (years) and *i* is the real rate of interest (%). The years to payback were estimated by calculating N when NPV becomes zero.

The initial and annual operating costs of the newly designed floating PV system was estimated in accordance with the initial investment cost standards of a PV system in the Czech Republic. Considering the actual size and tilt angle of the systems, typical values were used for supplying modules (c-Si technology) and electrical components [48], floats and anchoring [22], as well supporting structure (polygon configuration) and tracking mechanism. Total investment costs presented in Table 10 fit within the price range of real FPV systems of similar size around the globe [4], indicating reasonable assumptions.

**Table 10:** Parameters considered in financial assessment of the floating PV systems.

Parameters	Unit	FPV 35 deg (Tracking)	FPV 20 deg (Fix)
PV capacity	kW	253	323
Anchoring cost	EUR	29835	26887
Polygon structure cost	EUR	229336	-
Support elements	EUR	133162	118751
Rotation/reinforce rope cost	EUR	48696	10596
Average PV cost	EUR/kW	1000	1000
CAPEX	EUR/kW	2743	1484
O&M cost	%/year	0.2	0.2
PV degradation rates	%/year	0.5	0.5
Transmission losses	%	5	5
Avg. tariff inflation rate	%	1.8	1.8
Consumption (tracking)	MWh	8	-
Electricity to grid	MWh	313.2	333
Real interest rate	%	2.5	2.5
PV Lifecycle	years	30	30

**Figure 10:**
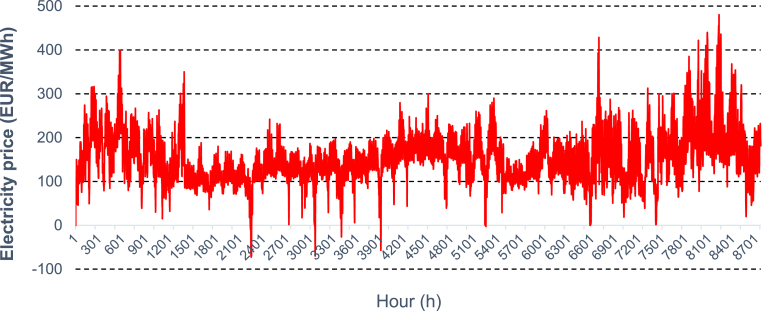
Hourly variation of electricity price used for the cost benefit of the generated electricity [50].

Incoming cashflows correspond to the savings on the electricity bill, or the benefit from supplying electricity to the grid. In this study, the most recent dynamic market prices (hourly) were used (see Figure 10) for the calculation of the annual income considering the annual energy output (kWh), 0.5%/year degradation rate (or it can be even higher due to high humid conditions [49]) for the selected PV technology and possible transmission & distribution losses. In the case of tracking systems, the consumption of the motor is also considered. Conversely, outcoming cashflows are the costs related to cleaning, electric revision and replacement of components given as a percentage of the initial installation costs (0.2%/year). Detailed values and assumptions made are presented analytically in Table 10.

**Figure 11:**
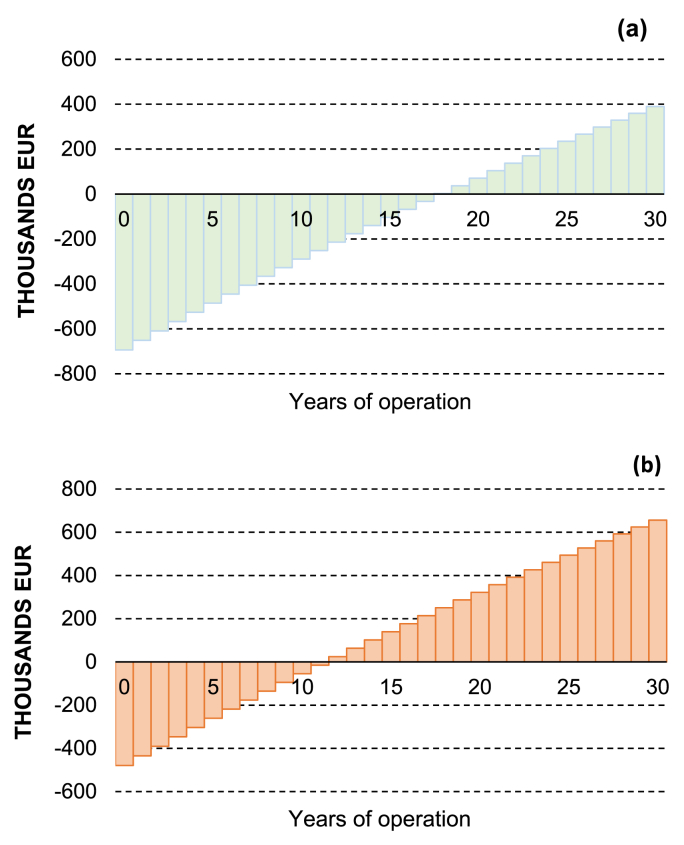
Net Present Value (NPV) associated with the investment of floating PV system (a) with and (b) without tracking mechanism, respectively, assuming flexible energy tariff.

Based on the parameters discussed, NPV was calculated over the lifetime of the system considering a real interest rate of 2.5% (10-year average for CZ) [51]. For the fixed system (20° tilt selected), actual costs, associated with year 0 in Figure 11b, lead to an investment of 479 thousand Euros. Despite the reduced PV capacity, investment costs can be considerably higher (¬44%) when a tracking system is assumed (Figure 11a), mainly due to the structural costs (Table 10).

From the results obtained in Figure 11a, increased investment of tracking system cannot be justified since it increases the discounted payback period (DPP) by 6 years compared to the fixed system (Table 11). On the other hand, indirect benefits (effective NPV) related to societal and environmental advantages, as well as land use reported in literature, can further increase the profitability/acceptability of the floating PV systems.

**Table 11:** Parameters considered in financial assessment of the floating PV systems.

Indicators	FPV 35 deg (Tracking)	FPV 20 deg (Fix)	
NPV (EUR)	389457	655946	
DPP (years)	18.8	12.3	

## 5 Conclusion

The present work provided an approach for the simulation and assessment of floating photovoltaic systems (FPVs). The effect of the most influencing parameters on the performance of the FPVs – such as module temperature, irradiance, albedo, PV modelling, panel spacing, tilt angle, as well as the benefits of including tracking mechanism – were taken into consideration using relevant models.

A simulation analysis of a floating PV system was performed under semi-continental climatic conditions of Czechia and key findings are given as follows:

- FPVs have around 3% higher energy gains and 5 °C lower module temperature compared to similar ground mounted PV systems.
- Low albedo effect for FPV system with high tilt angle compared to land-PV system (1% irradiance gain for 35° inclination).
- FPV systems with tracking mechanism and high tilt angles (≥30°) result in approximately 20% higher energy yield compared to fixed FPV installations.

In addition, two different large-scale FPV system designs were evaluated in terms of energy harvesting, installed capacity and economics. The first design corresponds to common fixed FPV systems installed in square (rectangular) floating platform, and the second design includes a vertical tracking mechanism installed in complex geometry (circular floating platform). The two scenarios were compared based on different tilt-angles and sizes. Despite the lower capacity, FPV with tracking system and high tilt angle had similar performance with a fixed system with low tilt angle. On the other hand, fixed PV system installation provided better feasibility with shorter payback period by almost 6 years.

Finally, it can be concluded that the presented approach can effectively be used for the simulation of FPVs, as demonstrated results were in a good agreement with some similar studies in literature. Given the immaturity and the complexity of the technology, further research is needed to generalize conclusions regarding the FPV gains, cooling mechanisms and floating structure effects. These should be studied separately in future works coupled with all the necessary experiments.

## Declarations

### Author contribution statement

Nikolaos Skandalos; Sofiane Kichou: Conceived and designed the experiments; Performed the experiments; Analyzed and interpreted the data; Contributed reagents, materials, analysis tools or data; Wrote the paper.

Petr Wolf: Contributed reagents, materials, analysis tools or data.

### Funding statement

This work has been supported by the Ministry of Education, Youth and Sports within the project CZ.02.1.01/0.0/0.0/15_003/ 0000464 Centre for Advanced Photovoltaics.

### Data availability statement

No data was used for the research described in the article.

### Declaration of interest's statement

The authors declare no conflict of interest.

### Additional information

No additional information is available for this paper.
